# Neuraminidase activity provides a practical read-out for a high throughput influenza antiviral screening assay

**DOI:** 10.1186/1743-422X-5-109

**Published:** 2008-09-26

**Authors:** Maryna C Eichelberger, Arash Hassantoufighi, Meng Wu, Min Li

**Affiliations:** 1 Center for Biologics Evaluation and Research, Food and Drug Administration, Bethesda, MD, USA; 2 High Throughput Biology Center and Department of Neuroscience, Johns Hopkins School of Medicine, Baltimore, MD, USA; 3 Division of Viral Products, OVRR, CBER, FDA; 8800 Rockville Pike, Building 29A 1D24; Bethesda MD, 20892, USA

## Abstract

**Background:**

The emergence of influenza strains that are resistant to commonly used antivirals has highlighted the need to develop new compounds that target viral gene products or host mechanisms that are essential for effective virus replication. Existing assays to identify potential antiviral compounds often use high throughput screening assays that target specific viral replication steps. To broaden the search for antivirals, cell-based replication assays can be performed, but these are often labor intensive and have limited throughput.

**Results:**

We have adapted a traditional virus neutralization assay to develop a practical, cell-based, high throughput screening assay. This assay uses viral neuraminidase (NA) as a read-out to quantify influenza replication, thereby offering an assay that is both rapid and sensitive. In addition to identification of inhibitors that target either viral or host factors, the assay allows simultaneous evaluation of drug toxicity. Antiviral activity was demonstrated for a number of known influenza inhibitors including amantadine that targets the M2 ion channel, zanamivir that targets NA, ribavirin that targets IMP dehydrogenase, and bis-indolyl maleimide that targets protein kinase A/C. Amantadine-resistant strains were identified by comparing IC_50 _with that of the wild-type virus.

**Conclusion:**

Antivirals with specificity for a broad range of targets are easily identified in an accelerated viral inhibition assay that uses NA as a read-out of replication. This assay is suitable for high throughput screening to identify potential antivirals or can be used to identify drug-resistant influenza strains.

## Background

Outbreaks of influenza account for much morbidity during winter months, and result in tens of thousands of deaths each year. The elderly and very young are particularly susceptible to more severe respiratory disease and death due to influenza. These individuals can be vaccinated but because the young are immunologically naïve, and the elderly are immunosenescent, vaccine preparations lack immunogenicity in these population groups [1-3]. Antivirals would clearly benefit these individuals and in addition would be of great value to the global population when no suitable vaccine is available to prevent infection [4]. This is likely the case when there is antigenic shift and a new virus strain emerges that could result in a world-wide pandemic. Pandemics that occurred in 1918, 1957 and 1968 were each the result of the transmission of influenza with a unique HA subtype, with the introduction of H1, H2 and H3 hemagglutinin (HA) gene segments from an avian virus source [5].

The avian H5N1 virus that is currently a pandemic threat has resulted in hundreds of human infections, with approximately 60% mortality rate. If such a strain becomes easily transmissible amongst people, there will be extensive death and disease unless a prophylactic vaccine is used or antivirals are administered. The only H5N1 vaccine licensed for emergency use in the United States contains inactivated A/Vietnam/1203/2004. There is no assurance that this vaccine will antigenically match the pandemic H5N1 strain, and so vaccine efficacy cannot be predicted. There is therefore a great need to stockpile effective antiviral drugs. Unfortunately, there are only two classes of antivirals that can be used to treat influenza; adamantanes that inhibit virus replication by blocking the influenza A M2 ion channel and neuraminidase (NA) inhibitors. Of these, the adamantanes are no longer effective against many recent influenza A virus strains [6,7] and most H5N1 strains are resistant to this class of drug [8]. Decreased sensitivity to the second class of antivirals that inhibit NA activity has been noted [9], and H1N1 viruses that are resistant to one of the two licensed NA inhibitors, oseltamivir, are prevalent in Europe [10].

In addition to problems associated with emergence of drug-resistant virus strains, each drug class has potential side effects. While the NA inhibitors were generally thought to have fewer toxic effects than amantadine and rimantadine, oseltamivir is no longer prescribed to children in Japan because of an association with neuropsychiatric disorders that include suicidal behavior, hallucinations and seizures [11]. Oseltamivir-induced delirium has also been reported in a geriatric patient [12]. There is clearly a need for licensure of additional inhibitors against influenza, particularly inhibitors to which resistant virus strains are less likely to emerge.

To fill this need, several new candidate antiviral agents have been identified [13]. In the process to select new candidates, methods targeted to a specific gene product or particular virus replication steps are commonly used; for example, viral RNA transcription [14]. However, assays that allow for identification of inhibitors with a broad range of targets increase the likelihood of obtaining a product that is effective. Unfortunately these latter viral inhibition assays are usually not suited to high throughput screening (HTS). In this report we describe modifications of the standard virus neutralization assay that facilitates its use in HTS. The key element to this assay is the use of viral NA as a means to quantify virus replication early after infection. This affords higher throughput with excellent signal/noise ratios, providing excellent assay sensitivity. In addition to presenting these properties, we use known influenza virus inhibitors to demonstrate the broad spectrum of antivirals detected by this assay.

## Results and discussion

### NA activity is a measure of influenza virus concentration

NA activity is required for release of newly formed virus particles from the infected cells [15] and consequently it is expected that all natural isolates of influenza A and B viruses have this enzyme activity. Approximately 50–100 NA molecules are incorporated into each virion and its activity has previously been used to quantify virus [16]. For the purpose of developing a high throughput screening assay, we needed to show that NA activity is readily measured in virus preparations after 1 hr incubation of virus with a fluorescent substrate, methyl-umbelliferyl-N-acetyl neuraminic acid (MU-NANA), and the read-out is proportional to the amount of virus present. The use of small chromagenic NA substrates has been appreciated for some time [17,18] and the use of MU-NANA substrate was first described in 1979 [19]. MU-NANA has subsequently been used to evaluate resistance of influenza to NA inhibitors [20].

**Figure 1:**
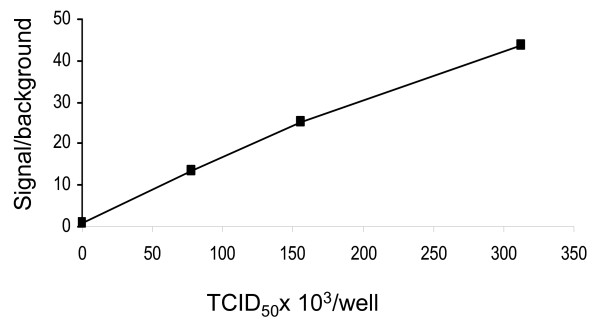
**NA activity can be used to quantify virus.** A/Wisconsin/67/2005 was serially diluted in PBS and 50 μl incubated with an equal volume of 20 μM MU-NANA for 1 hour at 37°C. Stop solution was added before reading fluorescence. The signal to background ratio at each concentration is shown. Under these conditions enzyme activity reaches a plateau with > 5 × 10^5 ^TCID_50 _due to limiting substrate.

We incubated different dilutions of virus with MU-NANA for 1 hour and measured relative fluorescence units (RFU) after addition of stop solution. The read-out (RFU) was directly proportional to the amount of virus added to each assay well (Figure 1). This confirmed the suitability of NA activity as a practical end-point index for development of a high throughput assay with rapid read-out for inhibition of virus replication. This "accelerated" virus inhibition (AVI) assay with NA as read-out was therefore called the "AVINA" assay.

### NA activity reflects replication of influenza in cells inoculated with a low virus dose

In the AVINA assay, a monolayer of cells is infected with virus in the presence or absence of inhibitor. MU-NANA is then added to the plate, or to supernatants from the plate, and the product of NA cleavage measured 1 hr later. This allows results to be obtained quickly and has the advantage that residual input virus is not detected since the inoculum dose (multiplicity of infection (MOI) of 0.01 to 0.02) is not detected under these conditions.

We established the assay conditions by comparing cell types, number of cells, medium, MOI, time of incubation with virus, time and temperature of incubation with NA substrate, and substrate concentration. Conditions that gave the greatest Z' scores (the confidence of identifying an inhibitor [21]), signal to background ratio and sensitivity were selected. A kinetic study that measured NA activity in supernatants collected at different time points after infection showed that while fairly good signals were obtained 16 hr post-infection, signal strength was increased and variability decreased when supernatants were harvested 20–22 hr post-infection (data not shown). Under these conditions, reproducible results were obtained within 24 hr of assay set-up.

**Figure 2:**
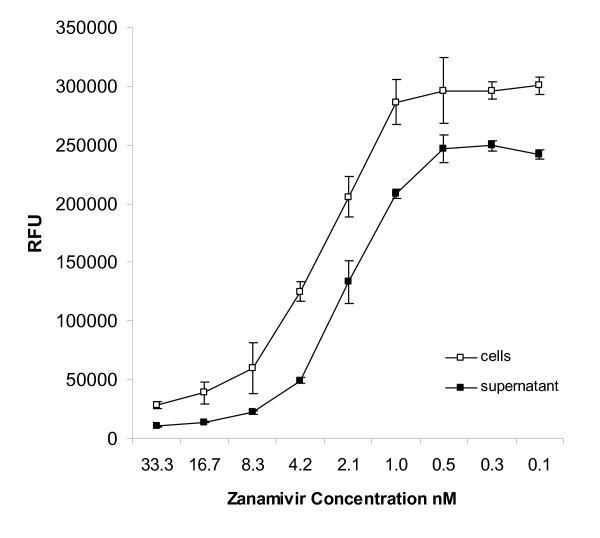
**NA activity in cell culture wells and in the supernatants of cells infected 20 hr earlier in the presence of different amounts of zanamivir.** In this experiment, MDCK cells were infected with 400 TCID_50 _A/Memphis/14/98 (MOI = 0.01). The inoculated amount of virus is not sufficient to measure NA activity at the selected conditions.

The primary inhibitors used to determine assay conditions suitable to identify antivirals were amantadine, an inhibitor of M2 ion channel activity [22] and zanamivir, an inhibitor of NA activity [23]. We showed inhibition by zanamivir and amantadine when cells were infected with influenza at a MOI between 0.01 and 0.1 for 16–24 hr. After incubation with virus, consistent results were obtained when the substrate was added to either the original MDCK-containing wells or the supernatants from infected cells. Figure. 2 shows data from an experiment that titrated zanamivir against A/Memphis/14/98. As expected, NA activity in cells with residual supernatant was greater than a small volume of supernatant alone (in the absence of inhibitor, relative fluorescence units (RFU) was 300,000 and 250,000 respectively). This difference reflects the additional activity of NA that is expressed on the surface of infected cells.

We hypothesized that measuring NA activity in supernatants would provide a more sensitive assay than quantifying NA activity in both cells and supernatants. When calculated from data shown in Figure 2, the 50% effective inhibition concentration (IC_50_) of zanamivir against A/Memphis/14/98 was 4.2 nM and 2.8 nM for assays that added substrate to the cell culture wells and cell supernatants, respectively. A similar increase in assay sensitivity was observed when IC_50 _of amantadine was calculated from the amount of NA activity in the supernatant. For A/Memphis/14/98, an IC_50 _> 33 μM was calculated for amantadine when NA activity of the total culture was quantified, while an IC_50 _of 1.2 μM was calculated when the amount of NA in the supernatant was measured. These differences support our hypothesis that measurement of NA activity in cell supernatants results in a more sensitive assay.

### The AVINA assay can be used with a broad spectrum of viruses and antivirals

Since NA is the read-out, viruses that are deficient in NA activity are not suitable for this assay. However, such NA-deficient viruses are not naturally selected and require addition of exogenous NA for in vitro growth. We successfully used the AVINA assay with a number of influenza A and B viruses. This included influenza A subtype H1N1 (A/PR/8/34 and A/New Caledonia/20/99), subtype H3N2 (A/Wuhan/359/95, A/Memphis/14/98 and A/Wisconsin/67/2005) and influenza B (B/Jiangsu/10/2003 and B/Malaysia/2506/2004).

The AVINA assay was largely established using A/Memphis/14/98 (H3N2) since it is sensitive to both amantadine and zanamivir, providing positive controls to optimize assay sensitivity. To evaluate the specificity of the assay, we determined the IC_50 _of zanamivir and amantadine against a number of different viruses. All viruses tested were sensitive to zanamivir; the IC_50 _of A/PR/8/34 (H1N1) was 3.6 nM, A/New Caledonia/20/99 (H1N1) was 3.0 nM, A/Memphis/14/98 (H3N2) was 2.8 nM. B/Jiangsu/10/2003 was less sensitive to inhibition by zanamivir, with an IC_50 _of 26.3 nM. Reduced sensitivity to zanamivir of Type B viruses in assays that measure inhibition of NA activity directly has been reported [20,24]. In these latter studies, the absolute IC_50 _values are similar but not identical to what we report, probably reflecting differences in the assays used: IC_50 _determined by inhibition of NA activity of whole virus does not measure the 'effective' inhibition of virus replication as is the case for our AVINA assay.

**Figure 3:**
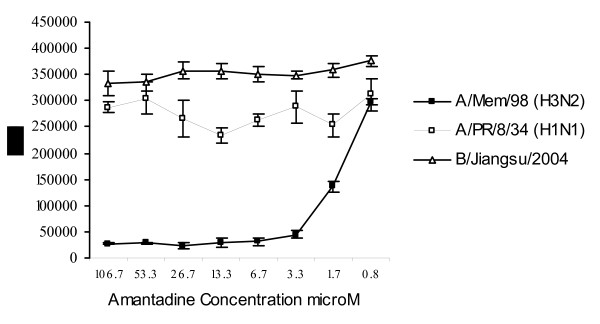
**Titration of amantadine against influenza A and B viruses in the AVINA assay.** Cells were infected with A/PR/8/34, A/Memphis/14/98 and B/Jiangsu/10/2003 at 0.01 MOI in the presence of serial dilutions of amantadine. The next day, NA activity was measured in the supernatant and IC_50 _calculated by GraphPad Prism software.

The AVINA assay correctly identifies virus sensitivity to amantadine (Figure 3). A/Memphis/14/98, showed good inhibition of replication in the presence of amantadine (IC_50 _is 1.2 μM). A/PR/8/34, a virus with known resistance to amantadine, was not inhibited by this drug (IC_50 _> 100 μM). As expected, influenza B viruses were resistant to amantadine.

**Figure 4:**
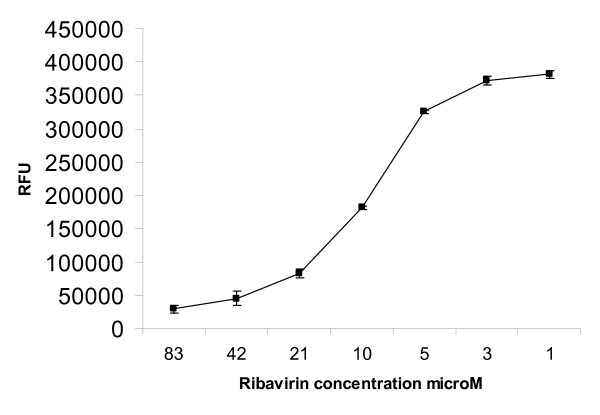
**Titration of ribavirin against A/Memphis/14/98 in the AVINA assay. **Cells were infected with 0.01 MOI A/Memphis/14/98 in the presence of serial dilutions of ribavirin. The next day, NA activity was measured in the supernatant and IC_50 _calculated by GraphPad Prism software.

To evaluate the breadth of antiviral targets that can be detected in the AVINA assay, we determined the IC_50 _for ribavirin, a broad spectrum antiviral that blocks viral replication [25], largely due to inhibition of IMP dehydrogenase [26], and bis-indolyl maleimide (BIM), an inhibitor of protein kinase A/C activity that is required for virus assembly [27]. Ribavirin is a nucleoside antimetabolite that protects mice from both lethal doses of influenza A and B viruses [25,28] but since results of clinical trials were mixed, this drug was not approved for use an anti-influenza agent in the USA [29]. In addition, ribavirin has severe side-effects: hemolytic anemia is observed, and it is a teratogen in some species. Oral ribavirin is however, available for treating influenza in Mexico (Vilona, ICN pharmaceuticals). In the AVINA assay, the IC_50 _of ribavirin for A/Memphis/14/98 was 10.8 μM (Figure 4).

**Figure 5:**
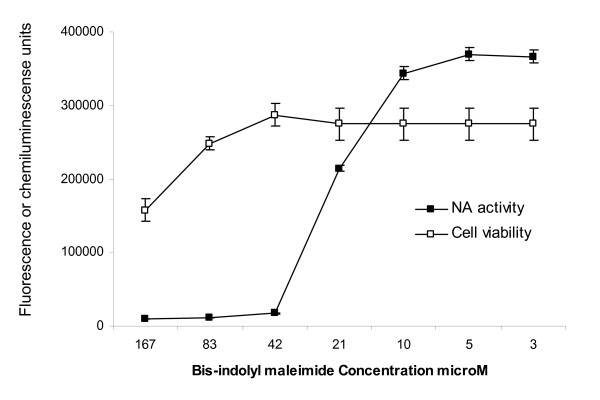
**Titration of bis-indolyl maleimide (BIM) against A/Memphis/14/98 in the AVINA assay.** Cells were infected with 0.01 MOI A/Memphis/14/98 in the presence of serial dilutions of BIM. The next day, NA activity was measured in the supernatant (shown as relative fluorescence units) and IC_50 _calculated by GraphPad Prism software. Cell viability was determined by ATPlite assay (shown as relative light units).

The protein kinases play a role in cellular functions that are required for successful influenza replication [30]. Inhibitors of protein kinase C such as BIM may prevent activation of ERK signaling that is necessary for export of the viral genome from the nucleus [27], or may have an effect on M2 ion channel activity since inhibition of cellular cation channel activity has been reported for BIM [31]. The IC_50 _of BIM measured by titration of the drug with A/Memphis/14/98 in the AVINA assay is 23 μM (Figure 5). This is a fairly high concentration (compared with IC_50 _of zanamivir and amantadine), raising concern that the observed inhibition is a result of its impact on cell viability and not a direct effect on virus replication. In preliminary experiments (data not shown) we demonstrated that cell viability in the AVINA assay can easily be determined by measuring ATP concentration in the culture (ATPlite assay, Perkin Elmer) after removal of a small volume of supernatant to assay NA activity. The cytotoxicity of each drug can therefore be evaluated in the same plate used to determine virus replication. There are some exceptions in which the stress response to a particular drug induces greater synthesis of ATP, resulting in data that is difficult to interpret. The relative light units (RLU) that reflect ATP levels at each BIM concentration are shown together with NA activity in Figure 5. BIM was not toxic to the cells at 23 μM but did show significant toxicity at 83 μM. While our results show specific inhibition of influenza virus replication by this protein kinase inhibitor, BIM is toxic to cells at doses that would need to be administered therapeutically, making it a poor antiviral candidate.

### The AVINA assay can be used in a high throughput format

**Figure 6:**
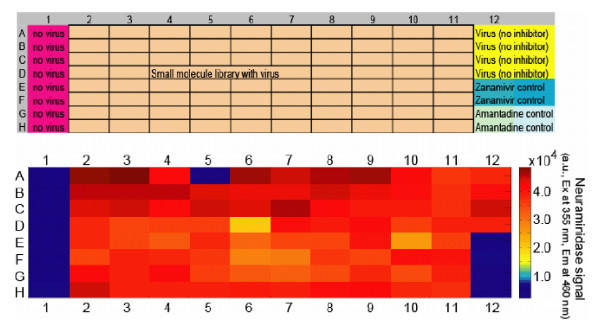
**Example of an HTS assay.** The plate map is shown in the upper panel, with wells set aside for background and virus controls. Each plate also includes known inhibitors, zanamivir and amantadine, at concentrations known to inhibit virus replication. The lower panel shows the results of NA activity for an assay that evaluated the antiviral activity of a panel of ion channel inhibitors. Activity is represented by a range of color; blue representing low relative fluorescence units (RFU), that is, no or little NA activity, and red representing high RFU, that is, a high amount of NA activity.

Final assay conditions were used in a blind screen of an ion channel inhibitor panel. The assay includes negative (quadruplicate wells that contained no inhibitor), and positive control wells (duplicate wells with 330 nM zanamivir or 33 μM amantadine), as shown in Figure 6. The results identified amantadine (Figure 6, well A5) and an amantadine derivative (Figure 6, well D6) as inhibitors of A/Memphis/14/98.

### The AVINA assay can be used to identify drug-resistant viruses

**Figure 7:**
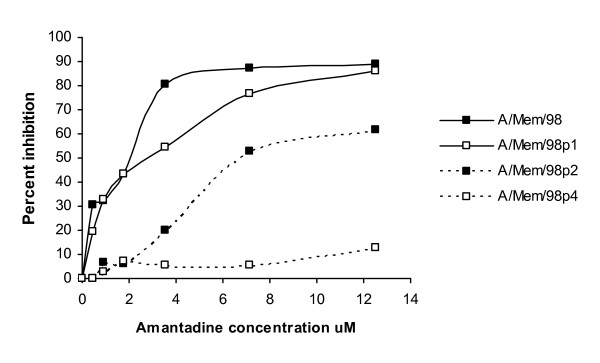
**Identification of amantadine-resistant virus preparations. **A/Memphis/14/98 was cultured in the presence of amantadine for one passage (A/Mem/98 p1), two passages (A/Mem/98 p2), or 4 passages (A/Mem/98 p4) in tissue culture. The TCID_50 _of each virus passage was determined, and 0.02 MOI added to serial dilutions of amantadine in the AVINA assay. The percent inhibition of virus replication is shown at each concentration of amantadine was calculated by: 100 × (average RFU at each amantadine concentration/average RFU in the absence of amantadine).

Culture of A/Memphis/14/98 in flasks of MDCK cells with 33 μM amantadine resulted in observable CPE after 2 days. Virus in this culture supernatant was passaged serially in the presence of amantadine and then the resultant supernatant was used to generate a virus stock (in the absence of inhibitor). After determining the TCID_50 _of each virus stock, an equivalent MOI of each virus preparation was used to infect MDCK cells in an AVINA assay that measured sensitivity to amantadine. Virus generated after a single passage in the presence of amantadine was slightly less sensitive to amantadine than the original stock; a 2^nd ^passage resulted in greater resistance and by the 4^th ^passage was completely resistant to amantadine (Figure 7). Our results demonstrate that even within a pool of viruses, the AVINA assay is capable of evaluating differences in sensitivity to viral inhibitors.

## Conclusion

In this report we describe the development of the AVINA assay, a high throughput assay that measures NA activity as a read-out for virus replication. The advantages of this assay are its ease of execution, reproducibility, and sensitivity to antivirals that target both early and late stages of replication with specificity for either viral or cellular targets. This assay can be applied to high throughput screening of chemical libraries to identify antivirals or can be used to determine IC_50 _and identify antiviral resistant virus strains.

## Methods

### Virus preparation and titration

Standard methods were used to prepare virus stocks in either MDCK cell cultures or embryonated chicken eggs and titrate these stocks on MDCK cells [32]. The viruses used were: tissue-culture adapted A/PR/8/34 (H1N1), A/New Caledonia/20/99 (H1N1), A/Memphis/14/98 (H3N2), A/Wisconsin/67/2005 (H3N2), and B/Jiangsu/10/2003. Briefly, a confluent layer of MDCK cells was washed in serum-free medium and inoculated with virus at a 0.001 MOI. After 3 days the preparation was centrifuged and the supernatant aliquoted and stored at -70°C. Ten day old embryonated chicken eggs (CBT Farms, Chestertown, MD) were inoculated with virus and incubated for at least 48 hr. After overnight chilling at 4°C, the allantoic fluid was harvested, cellular debris pelleted and virus aliquots stored at -70°C. The infectious titer of virus was determined by ten-fold serial dilution and inoculation of MDCK cells in quadruplicate wells of a 96-well plate. Cytopathic effect indicated the presence of virus, with the TCID_50 _end-point defined as the inverse of the dilution that showed CPE in 50% of the wells.

### Antivirals

Amantadine (Sigma Chemicals, St Louis MO) was dissolved in DMSO and then diluted to make a 2 mM stock solution in PBS. Zanamivir (Relenza, Roche) 5 mg caplet was dissolved in PBS to make a 2 μM solution. Stock solutions (2 mM) of ribavirin (Sigma, St Louis, MO) and bis-indolyl maleimide (BIM, Sigma) were prepared in PBS. All stock solutions were aliquoted and stored at -20C.

### Accelerated Viral Inhibition Assay

MDCK cells were washed in serum-free medium (EMEM containing glutamine and penicillin and streptomycin) and 50 μl aliquoted into flat-bottomed 96-well plates at 8 × 10^5^/ml. In assays to define conditions, the cells were allowed to adhere overnight before addition of 50 μl controls (known antiviral agents or diluent (serum-free medium)); 50 μl virus containing 0.02 MOI, followed by 50 μl EMEM containing 3% bovine serum albumin (BSA) and TPCK-treated trypsin (5 μg/ml). However, assay reproducibility and sensitivity was retained when antivirals, virus, BSA and TPCK-trypsin were added simultaneously, and therefore the results shown in this report used our standard conditions: antivirals (or dilutions of antivirals) we made in 30 μl serum-free EMEM followed by the addition of 90 μl virus diluted in EMEM containing BSA (1%) and TPCK-treated trypsin (5 mg/ml). A portion (100 μl) of this mixture was added to wells containing MDCK cells plated the previous day in serum-free medium. The assay can also be performed without prior establishment of a cell monolayer, with washed cells added directly to the antiviral/virus mixture. Twenty hrs after incubation at 37°C in 5% CO_2_, 25 μl of the supernatant was harvested for NA assay as described below. Viability of cells in the culture plate was determined by ATPlite assay (PerkinElmer) following the manufacturer's instructions.

### Neuraminidase assay

Cell supernatants (25 μl) were transferred to a black 96-well plate and 75 μl of 20 μM MU-NANA added. After incubation of the plate at 37°C for 1 hr, 100 μl stop solution (0.1 M glycine, pH 10.7–25% EtOH) was added to each well and fluorescence read on a Victor V (Perkin Elmer) with excitation and emission filters of 355 nm and 460 nm respectively.

### Statistical analysis

Statistical analysis was performed using GraphPad Prism software, with sigmoidal non-linear curves used to calculate the IC_50 _of inhibitors.

## List of abbreviations

AVINA: Accelerated Virus Inhibition assay, with NA as read-out; BIM: bis-indolyl maleimide; BSA: bovine serum albumin; CPE: cytopathic effect; ID_50_, 50% inhibition dose; HA: hemagglutinin; HTS: high-throughput screening; MOI: multiplicity of infection; MU-NANA: methyl-umbelliferyl-N-acetyl neuraminic acid; NA: neuramindase; RFU: relative fluorescence units; RLU: relative light units.

## Declaration of competing interests

The authors declare that they have no competing interests.

## Authors' contributions

MCE designed and supervised experiments, MW performed experiments and statistical evaluation of results, AH performed experiments and analyzed results and ML designed and supervised experiments. All authors contributed to writing or revision of the manuscript.
